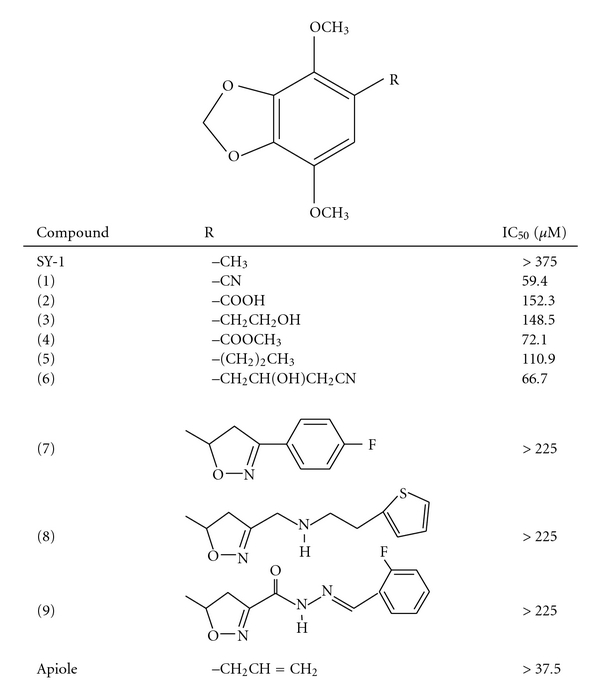# Study of the Anti-Proliferative Activity of 5-Substituted 4,7-Dimethoxy-1,3-Benzodioxole Derivatives of SY-1 from *Antrodia camphorata* on Human COLO 205 Colon Cancer Cells

**DOI:** 10.1093/ecam/nep230

**Published:** 2011-05-03

**Authors:** Hsiu-Man Lien, Po-Tsun Kuo, Chao-Lu Huang, Jung-Yie Kao, Ho Lin, Ding-Yah Yang, Ya-Yun Lai

**Affiliations:** ^1^Department of Chemistry, Tunghai University, Taichung, Taiwan; ^2^Yusheng Biotechnology Co. Ltd., Taichung, Taiwan; ^3^Institute of Biochemistry, College of Life Science, National Chung Hsing University, Taichung, Taiwan; ^4^Department of Life Science, National Chung Hsing University, Taichung, Taiwan; ^5^Department of Applied Chemistry, Chung Shan Medical University, No. 110, Sec. 1, Jianguo N. Rd., Taichung 402, Taiwan; ^6^Department of Biochemistry, Chung Shan Medical University Hospital, Taichung, Taiwan

## Abstract

A set of 10 4,7-dimethoxy-1,3-benzodioxole derivatives based on a lead compound previously discovered by our group, SY-1, which was isolated from *Antrodia camphorata*, were evaluated for their *in vitro* inhibitory activity on human colorectal carcinoma cells (COLO 205). Structure-activity relationship studies of the 10 compounds indicated the importance of the chain length of the alkyl group at the 5-position, and the 2-propenyl substituent named “apiole” exhibited the most potent inhibitory activity. In the present study, we demonstrate that the SY-1 analogue “apiole” decreased the proliferation of COLO 205 cells, but not that of normal human colonic epithelial cells (FHC). The G0/G1 cell cycle arrest induced by apiole (75–225 **μ**M) was associated with significantly increased levels of p53, p21 and p27 and decreased levels of cyclin D1. Concerning COLO 205 cell apoptosis, apiole (>150 **μ**M) treatment significantly increased the levels of cleaved caspases 3, 8, 9 and bax/bcl-2 ratio and induced ladder formation in DNA fragmentation assay and sub-G1 peak in flow cytometry analysis. These findings suggest that apiole can suppress COLO 205 cell growth; however, the detailed mechanisms of these processes require further investigation.

## 1. Introduction

The most prevalent form of cancer among Taiwanese people is colorectal cancer, topping the list of the five most common cancers in Taiwan according to a 2006 cancer report released by the Department of Health. About 15.03 per 100 000 individuals die per year of colorectal cancer, and the current clinical therapies are limited to surgery, radiotherapy, general chemotherapy and gene therapy [1–3]. There is clearly an urgent need to identify new therapeutic agents for the treatment of colon cancer.

*Antrodia camphorata* has been used in traditional Chinese medicine to treat hypertension, pruritus, diarrhea and liver cancer. Previously, we reported that a unique compound, 4,7-dimethoxy-5-methyl-1,3-benzodioxole (SY-1), isolated from *A. camphorata* extracts, was able to profoundly decrease the proliferation of human COLO cancer cells (COLO 205) through G0/G1 cell cycle arrest and the induction of apoptosis [4]. Although SY-1 caused a significant increase in p53 expression in COLO 205 cells, we decided to evaluate the possibility of further modifications at position 5 of the SY-1 structure, with the goal of developing more efficient anti-cancer agents. The anti-tumor activities of 4,7-dimethoxy-1,3-benzodioxoles are largely unknown and therefore we screened 10 5-substituted 4,7-dimethoxy-1,3-benzodioxoles as model agents to investigate novel anti-human colon cancer agents with increased potency. Thus, a series of 4,7-dimethoxy-1,3-benzodioxole derivatives were evaluated for their anti-proliferation activity.

Among those compounds, 4,7-dimethoxy-5-(2-propen-1-yl)-1,3-benzodioxole, also named apiole, apiol or parsley apiol, which is an essential oil component of the fruits of *Petroselinum crisp* [5], seeds of *Enterolobium contortisiliquum* (leguminosae) [6], wild-growing *Salvia aegyptiaca* [7], leaves of *Cinnamomum verum Presl.* [8] and leaves and fruits of Caraway (*Carum carvi L.*) [9] and *Pituranthos chloranthus ssp.* [10], was evaluated. This compound shows potent calcium channel blocking activity [5, 11], with a strong depressing effect on the force of contraction (IC_50_ = 30 *μ*M). Essential oil from seeds of *Enterolobium contortisiliquum* that contained apiole shows weak antimicrobial activity against gram-positive bacteria (*Bacillus subtilis, Bacillus cereus, Staphylococcus aureus, Micrococcus luteus*) [6]. This report is the first to demonstrate that apiole could have more potent inhibitory effects than SY-1 against COLO 205 and could be developed into a potent anti-colon cancer drug.

## 2. Methods

### 2.1. Chemicals and Reagents

5-Substituted 4,7-dimethoxy-1,3-benzodioxoles (compounds 1–9) were purchased from Aurora Fine Chemicals Ltd, Austria, and apiole was obtained from the Uni-onward Company, Taiwan. The structures of all compounds were identified by NMR spectroscopy.

### 2.2. Cell Culture

The COLO 205 (p53 wild-type) cell line was isolated from human colon adenocarcinoma (CCL-222; American Type Culture). The cell line was grown in RPMI-1640 medium (Invitrogen, Carlsbad, CA) containing 10% fetal bovine serum (FBS) (Invitrogen Carlsbad, CA) and 1% penicillin–streptomycin (Invitrogen, Carlsbad, CA). FHC (CRL-1831; American Type Culture Collection) is a cell line derived from long-term epithelial cell cultures of normal human fetal colonic mucosa [12]. Cultures were incubated in a 37°C incubator with a humidified atmosphere of 5% CO_2_.

### 2.3. Cell Viability Assay

The anti-proliferative effects of the compounds on cultured cells were measured using the MTT (3-(4,5-dimethylthiazol-2-yl)-2,5-diphenyltetrazolium bromide) assay. The cells were seeded at a density of 2 × 10^4^ cells per well in 24-well culture plates overnight and then treated with various concentrations of the compounds. After 24, 48 and 72 hours of incubation, 30 *μ*L of 2 mg mL^−1^ MTT solution (in phosphate buffered saline (PBS), pH 7.4) was added to each well, and the plate was incubated for another 3 hours. Following incubation, the culture medium was removed from the wells by slow aspiration and replaced with 200 *μ*l DMSO. The absorbance of each well was measured using an enzyme-linked immunosorbent assay (ELISA) reader at 550 nm.

### 2.4. Flow Cytometry Analysis

The COLO 205 cells were harvested at various times after treatment with different concentrations of apiole with trypsin–EDTA, washed twice with filtered PBS and then fixed in 70% ethanol at 4°C for >24 hours. Fixed cells were washed once with filtered PBS and treated with DNase-free RNase (2 U mL^−1^) for 30 min, followed by staining with propidium iodide reagent (3.8 mM sodium citrate, 0.1% Triton X-100 and 20 *μ*g mL^−1^ propidium iodide). The DNA content was measured by flow cytometry (Cytomics FC500, Beckman Coulter). The percentage of cells in each phase of the cell cycle was analyzed using software (Multicycle 32bit version, Beckman Coulter).

### 2.5. Immunoblotting Analysis

The COLO 205 cells were treated with various concentrations of apiole for different durations, and the cell lysates produced in the lysis buffer (20 mM Tris–HCl, pH 7.4, 1% NP-40, 137 mM NaCl, 50 *μ*M EDTA, protease inhibitor cocktail and 1 mM PMSF) were used for immunoblotting [13]. Protein samples were analyzed by direct immunoblotting (100 *μ*g per lane). Immunodetection was carried out by probing with appropriate dilutions of specific antibodies at 4°C overnight. The primary antibodies were diluted at 1 : 100 and 1 : 1000, including anti-cyclin D1 and anti-p27 (Santa Cruz, Inc., CA, USA), anti-actin (Chemicon Co., MA, USA), anti-cleaved caspase 3,8,9 (Cell Signaling Technology, Inc., MA, USA), anti-p21 and anti-p53 (BD Bioscience, CA, USA), anti-bax and anti-bcl-2 (Assay Designs, MI, USA). Secondary antibodies, including peroxidase-conjugated goat anti-mouse or anti-rabbit antibodies and alkaline phosphatase-conjugated goat anti-mouse antibody (Jackson ImmunoResearch Laboratory, PA, USA), were incubated at room temperature for 1 hour at a dilution of 1 : 5000. ECL detection reagent and BCIP/NBT substrate solution (Perkin Elmer Co., MA, USA) were used to visualize immunoreactive proteins on PVDF membranes after transfer by Trans-Blot SD (Bio-Rad Co., CA, USA).

### 2.6. Analysis of DNA Fragmentation

Apoptosis in COLO 205 cells subjected to the various treatments was determined by DNA fragmentation analysis [14]. The genomic DNA was extracted and electrophoresed in a 2% agarose gel. The DNA was visualized by ethidium bromide staining.

### 2.7. Statistics

All values are presented as means ± SE. Significant comparisons were subjected to Dunnett's one-way ANOVA. Significance was defined as *P* < .05.

## 3. Results

### 3.1. Cytotoxic Activity of 5-Substituted 4,7-Dimethoxy-1,3-Benzodioxole Derivatives on Human Colon Cancer Cells

**Figure 1:**
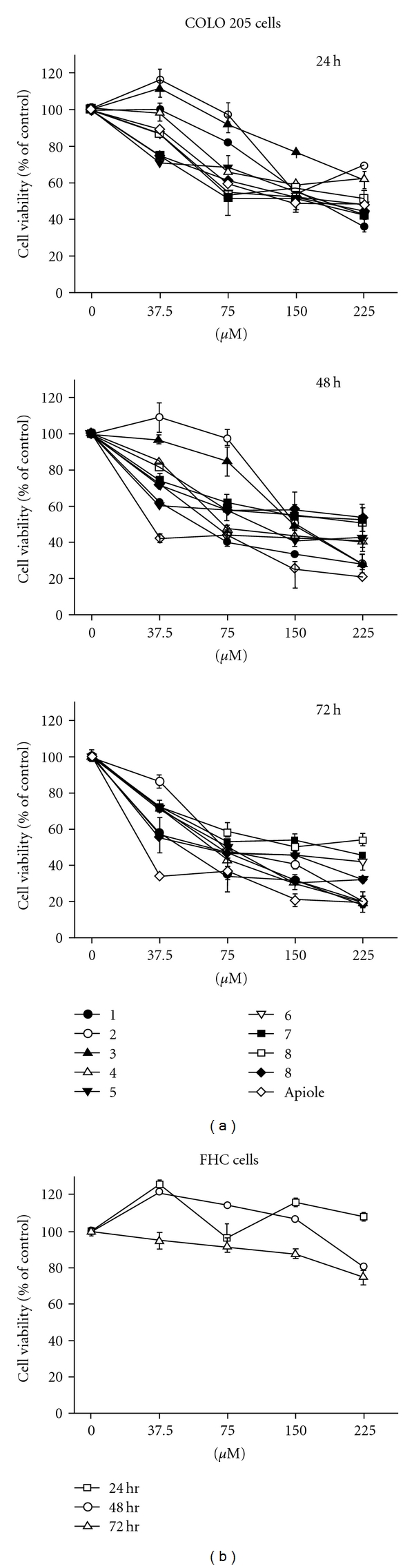
Cell growth inhibitory effects of SY-1 derivative compounds in normal and malignant human cells. (a) Human colorectal adenocarcinoma cells (COLO 205) were treated with SY-1 derivatives at varying doses and for different durations. (b) FHC cells were treated with apiole (37.5–225 *μ*M) for varying durations. An MTT assay was then performed to determine the cell viability. All of these experiments were performed at least three times, and the results are presented as means ± SE.

Our previous studies showed that SY-1 induces cytotoxicity in the human colorectal adenocarcinoma COLO 205 cell line [4]. In the present study, anti-proliferation effects of 10 SY-1 derivatives were examined using the MTT assay. COLO 205 cells were treated with each compound (37.5–225 *μ*M) for varying durations. Similar anti-proliferation activity was observed in the COLO 205 cell line (Figure 1(a)). Interestingly, we found that 5-position functional groups with aliphatic substituents (compounds 4, 5 and apiole) had more potent anti-proliferative effects than polar alkyl functional groups (compounds 1–3). The IC_50_ values of compounds 1, 2, 3, 4, 5 and 6 were measured to be 59.4, 152.3, 148.5, 72.1, 110.9 and 66.7 *μ*M, respectively. However, bulkier substituents at the 5-position (such as compounds 7, 8 and 9) displayed very weak (>225 *μ*M) inhibitory activity (Figure 1(a)). 

The most potent compound among 10 4,7-dimethoxy-1,3- benzodioxoles against COLO 205 was apiole, which exhibited IC_50_ values that were significantly <37.5 *μ*M at 48 hours and 72 hours (Figure 1(a)). The side chain derivatives were divided into 2-propenyl (apiole) and *n*-propyl groups (compound 5), and the anti-proliferative activity of apiole was found to be 3-fold higher than that of *n*-propyl in COLO 205 cells. To test whether the suppression of COLO 205 cancer cell proliferation by SY-1 derivatives was cell-type specific, normal human colonic epithelial (FHC) cells were treated with apiole at varying doses and for different durations (Figure 1(b)). The cytotoxic activity of apiole was not observed in the normal human cell line (Figure 1(b)). Accordingly, apiole was selected as a more potent compound to study the mechanism of its anti-proliferative activity compared to the other SY-1 derivatives with structural modifications at position 5 of 4,7-dimethoxy-1,3-benzodioxole.

### 3.2. Apiole Induces Cell Cycle Arrest in Human Colon Cancer Cells

**Figure 2:**
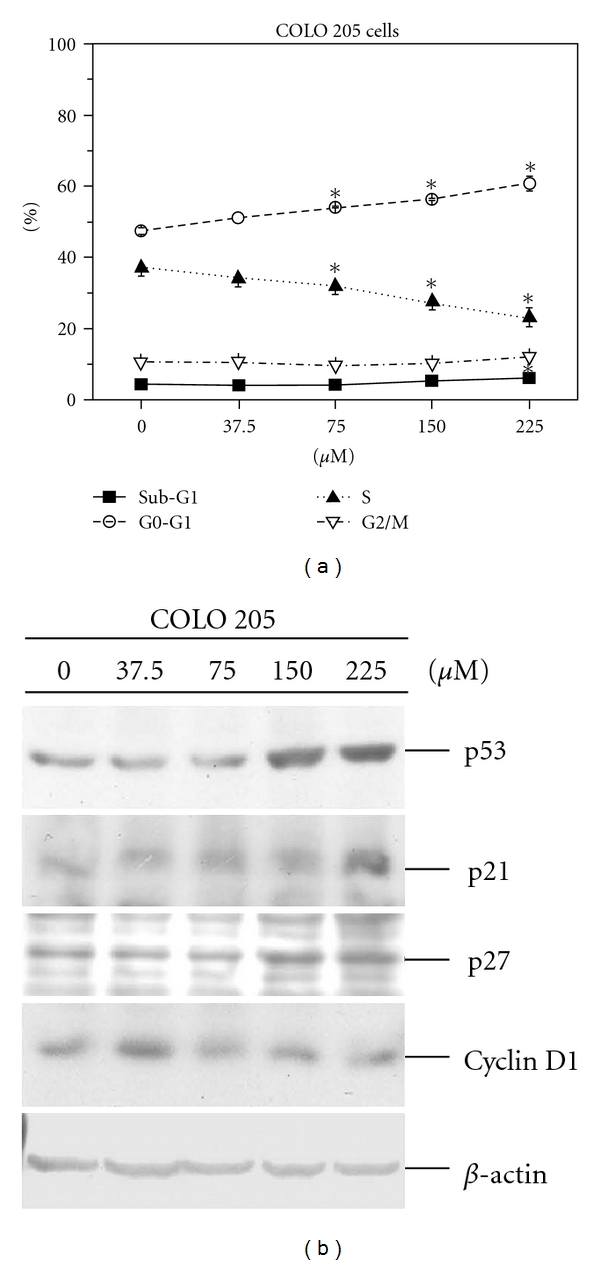
Dose-dependent effects of apiole on G0/G1 phase arrest in COLO 205 cells. COLO 205 cells were treated with apiole (0, 37.5, 75, 150 and 225 *μ*M) in 0.05% DMSO and sampled by flow cytometry and immunoblotting analysis 15 hours following treatment. (a) The percentages of cells in the sub-G1, G0/G1, S and G2/M phases of the cell cycle were determined using Multicycle analysis software. Three samples were analyzed in each group, and the values represent means ± SE. *P < .05 versus the control group. (b) Effect of apiole on the expression levels of cell cycle regulatory proteins. Protein extracts (100 *μ*g per lane) were separated by SDS-PAGE and probed with specific antibodies. Membranes were also probed with an anti-*β*-actin antibody to correct for differences in protein loading.

Figure 2 demonstrates the dose-dependent effects of apiole on G0/G1 arrest. According to our previous studies [4, 15], the greatest difference between COLO 205 cells in the G0/G1 cell population of the treatment and control groups was recorded 15 hours after replacement with complete medium. Accordingly, this time point (15 h) was selected to study the dose-dependent effects of apiole on the induction of cell cycle arrest, as determined by flow cytometry analysis. As shown in Figure 2(a), significant G0/G1 arrest was induced in COLO 205 cells treated with apiole (>75 *μ*M), and this effect was dose dependent. Our previous study showed that cyclin-dependent kinase inhibitors (CDKi), such as p21 and p27, are upregulated in human colon cancer cells arrested in G0/G1 by treatment with anti-cancer agents [4, 15, 16]. In the present study, COLO 205 cells exposed to apiole (<150 *μ*M) displayed induction of p21, p27 and p53 and decreased expression of cyclin D1 (Figure 2(b)). 

### 3.3. Apiole Activates Caspases for Apoptosis in Human Colon Cancer Cells

**Figure 3:**
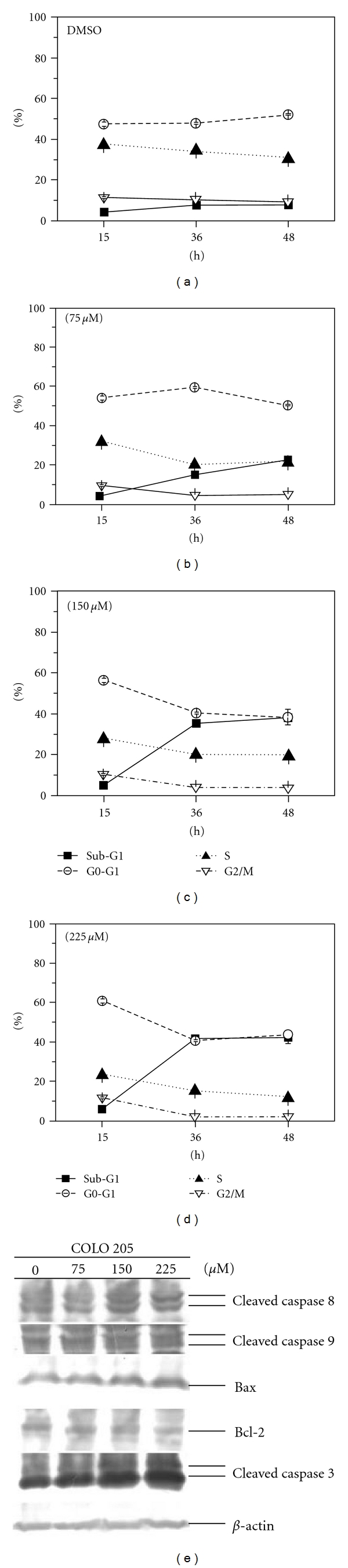
Time- and dose-dependent effects of apiole on the apoptosis of COLO 205 cells. Synchronized COLO 205 cells were sampled immediately at 15, 36 and 48 hours after 10% FCS treatment. Cells in the (a) control, (b) 75 *μ*M, (c) 150 *μ*M and (d) 225 *μ*M apiole-treated groups were then harvested for flow cytometry analysis. The percentages of cells in the sub-G1, G0/G1, S and G2/M phases of the cell cycle were determined using the well-known Multicycle analysis software. Three samples were analyzed in each group, and the values represent means ± SE. *P < .05 versus the control group. (e) Effect of apiole treatment on apoptosis regulatory proteins in human COLO 205 cells. The COLO 205 cells were treated with the same regimens as described in ((a–d)). Protein lysates were separated by SDS-PAGE and probed with specific antibodies. Membranes were also probed with an anti-*β*-actin antibody to correct for differences in protein loading.

**Figure 4:**
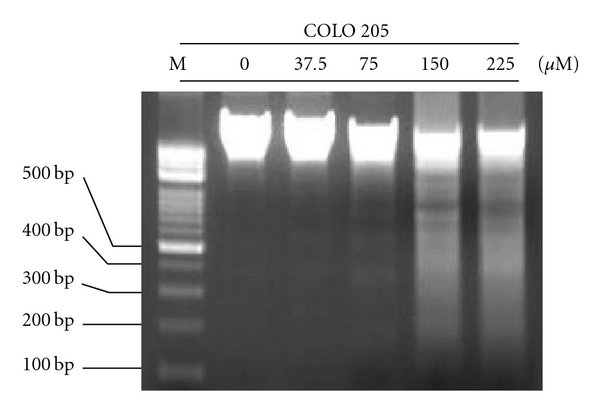
Dose-dependent apiole-induced DNA fragmentation in human colon cancer. COLO 205 cells were treated with apiole at the indicated doses. Induction of apoptosis in all cells was shown by DNA fragmentation using electrophoresis of genomic DNA. DNA fragmentation was examined 36 hours after drug treatment.

To further demonstrate that apoptosis could be detected in human COLO 205 cells, the cells were exposed to apiole for 36–48 hours. Figures 3(a)–3(d) demonstrates the time- and dose-dependent effects of apiole on cell cycle regulation. A higher dose (150, 225 *μ*M) of apiole at 36 and 48 hours significantly increased the population of the sub-G1 peak, as detected by flow cytometry analysis. Previous work has demonstrated that the occurrence of apoptosis requires the activation of caspases [17, 18]; therefore, we investigated the involvement of caspase activation and bax/bcl-2 ratio in COLO 205 cells following apiole treatment by immunoblotting analysis. The results show that apiole (150 or 225 *μ*M for 36 h) induced apoptosis in COLO 205 cells with the increase of bax/bcl-2 ratio and cleavage of caspases 3, 8 and 9 (Figure 3(e)). Next, we investigated apiole-induced apoptosis on COLO 205 cells by DNA fragmentation analysis. DNA fragmentation was only observed in the apiole-treated COLO 205 cells after 36 hours (>75 *μ*M). Indeed, DNA fragmentation was induced by a higher dose of apiole (150 *μ*M) as shown in Figure 4. These results suggest that apiole induced apoptotic cell death in the COLO 205 cells. 

## 4. Discussion

The present study was to investigate the cytotoxic effects against human COLO 205 colon cancer cells and anticancer mechanisms of 10 4,7-dimethoxy-1,3-benzodioxole derivatives of SY-1 isolated from dried fruiting bodies of *A. camphorata* (Table 1). Our previous study is the first demonstration that SY-1 inhibits human colon cancer cell proliferation through arrest of the cell cycle and activation of the cellular apoptosis response [4]. However, other derivatives with the structural skeleton of 5-substituted 4,7-dimethoxy-1,3-benzodioxoles were still not found anticancer activities yet. Thus, we tried to study anti-proliferative effects on COLO 205 colon cancer cell of the 5-substituted derivatives varied with cyano (−CN), carboxylic (−COOH), 2-hydroxyethyl (−CH_2_CH_2_OH), methyl ester (−COOCH_3_), propyl (−CH_2_CH_2_CH_3_), 2-propen-1-yl (−CH_2_CH = CH_2_) and three aromatic side chain groups. Structure–activity relationships among the 10 5-substituted 4,7-dimethoxy-1,3-benzodioxole compounds derived from SY-1 (Figure 1) showed that apiole is the most potent compound against COLO 205 cells. However if the modification on 5-position is substituted with an *n*-propyl (compound 5), other than allyl (apiole), compound 5 shows very weak anti-proliferative activity on COLO 205 cells. In 1984 Phillips et al. discovered that apiole have only very low adduct-forming ability *in vivo* [19]. Moreover, apiole is phytotoxic to the monocots and are weakly antifungal and is not toxic to the MIB-producing cyanobacterium *O. perornata* and the green alga *S. capricornutum* [20]. To our knowledge, our study in the article is the first finding that apiole has anti-proliferation effect via G0/G1 phase in COLO 205 cells. 

Apiole showed the most efficient suppression of COLO 205 cell proliferation and excitedly was less cytotoxic to normal colonic FHC cells (Figure 1). p53 is a tumor suppressor protein that has been correlated with cell cycle arrest [21]. Cell cycle progression is regulated by proteins downstream of p53, including p21, p27 (CDKi) and cyclin D1 [22–24]. A recent study demonstrated that knockdown of mutant p53 enhances the sensitivity of human colon tumor cells to the growth suppression caused by various chemotherapeutic drugs [25]. In the previous study, SY-1 inhibited colon cancer cell growth in the G0/G1 phase and induced apoptosis and colony formation in COLO 205 cells (wild-type p53), but not in HT 29 (mutant p53) cells [4]. Apiole is an SY-1 analogue, and the present study showed that it can inhibit the growth of COLO 205 cells more profoundly than SY-1 treatment. By flow cytometry analysis, apiole at a concentration of 75 *μ*M was found to induce G0/G1 cell cycle arrest of COLO 205 cells (Figure 2(a)). With regard to related cell cycle proteins, our data demonstrate that p53 and its regulatory gene, p21 and p27, were transcriptionally upregulated, which in turn mediated apiole-induced G0/G1 arrest in COLO 205 cells by inhibiting cyclin D1 (Figure 2(b)).

**Figure 5:**
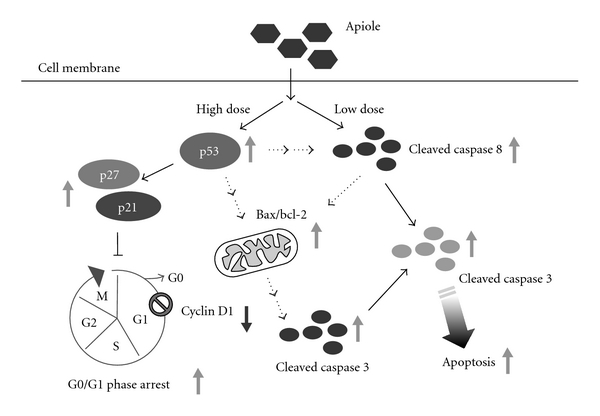
Schematic diagram of signaling pathways involved in apiole-induced cell-cycle arrest and apoptosis in human COLO 205 cells.

The recent study further demonstrated that the cytosolic p53 directly activated the proapoptotic bax protein [26]. Our study demonstrated that apiole induced p53 and bax protein expression Figure 5. Based on these findings, we propose that when p53 accumulates in the cytosol, it can function analogously to the subset of proapoptotic bcl-2 protein to activate bax and trigger the apoptosis mediated by mitochondria-signaling processes. In the study, cleavage of caspase 8 was detected in COLO 205 cells in response to a lower dose (75 *μ*M) of apiole. In contrast, proapoptotic bax, bcl-2 proteins and cleavage of caspase 9 were detected only at a higher dose (>150 *μ*M). The sub-G1-phase population of COLO 205 cells was only detected 36 hours after apiole (>150 *μ*M) treatment (Figures 3(a)–3(d)). The extrinsic caspase 8 pathway and the intrinsic mitochondrial pathway are the two major signaling pathways that regulate apoptosis. Activation of bid protein by caspase 8 links the extrinsic and intrinsic apoptotic pathways through mitochondrial damage [27]. Our data show that apiole-induced apoptosis in COLO 205 cells occurred via the mitochondrial (caspase 9) pathway and the caspase 8 pathway, with the possible link of bid protein activity between the two pathways. A molecular marker for apoptosis, cleaved caspase 3, increased immediately prior to COLO 205 cell death. In conclusion, apiole appears to be a universal inhibitor of cancer cell proliferation via induction of G0/G1 phase cell cycle arrest and apoptosis through activation of p53 signaling in the COLO 205 human colon cancer cell line. These observations suggest that apiole could be used as a novel treatment in cancer chemotherapy. 

## Funding

National Science Council of R.O.C. (grant NSC 96-2320-B-040-010 to Y.-Y. L.). Chung Shan Medical University, Taichung, Taiwan (grant CSMU 94-OM-B-006 to Y.-Y. L.)

## Figures and Tables

**Table 1:** Structures and IC_50_ values of 5-substituted 4,7-dimethoxy-1, 3-benzodioxole derivatives for the inhibition of COLO 205 cells at 48 hours.