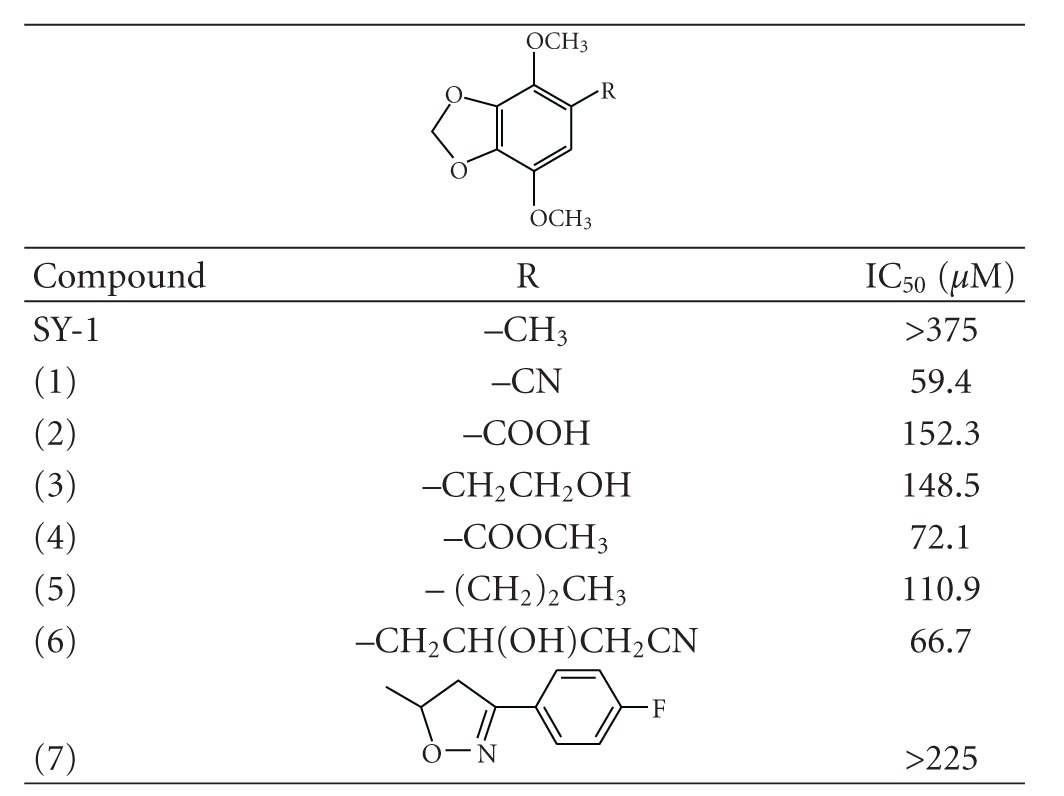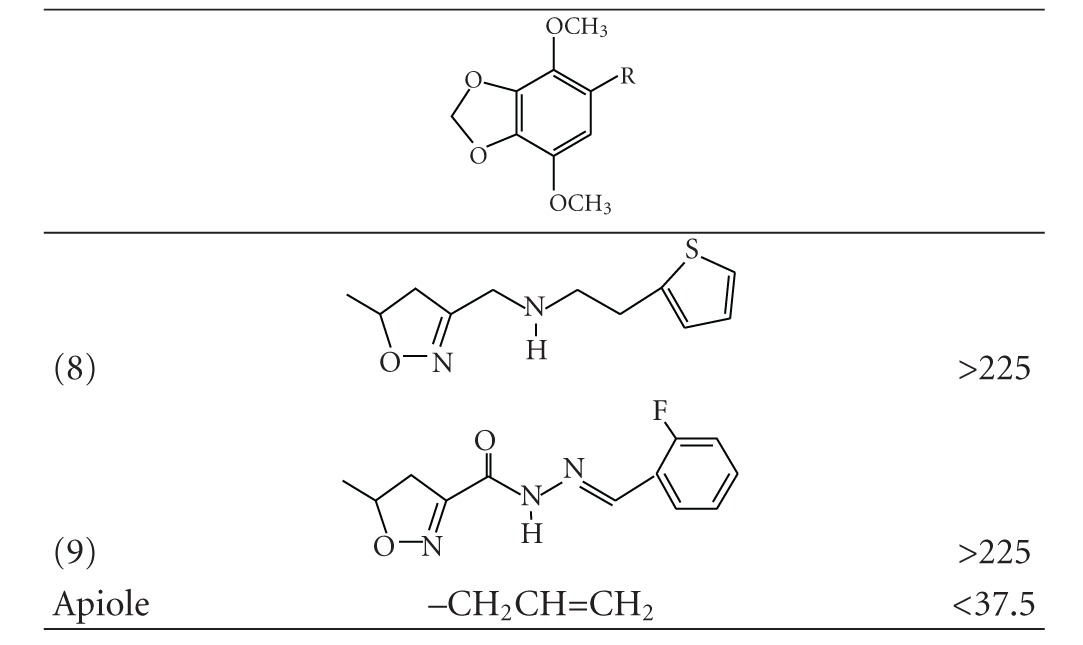# Erratum to “Study of the Anti-Proliferative Activity of 5-Substituted 4,7-Dimethoxy-l,3-Benzodioxole Derivatives of SY-1 from *Antrodia camphorata* on Human COLO 205 Colon Cancer Cells”

**DOI:** 10.1155/2012/148127

**Published:** 2012-07-29

**Authors:** Hsiu-Man Lien, Po-Tsun Kuo, Chao-Lu Huang, Jung-Yie Kao, Ho Lin, Ding-Yah Yang, Ya-Yun Lai

**Affiliations:** ^1^Department of Chemistry, Tunghai University, Taichung, Taiwan; ^2^Research and Development Department, Yusheng Biotechnology Co. Ltd., Taichung, Taiwan; ^3^Institute of Biochemistry, College of Life Science, National Chung Hsing University, Taichung, Taiwan; ^4^Department of Life Science, National Chung Hsing University, Taichung, Taiwan; ^5^Department of Applied Chemistry, Chung Shan Medical University, Taichung, Taiwan; ^6^Department of Biochemistry, Chung Shan Medical University Hospital, Taichung, Taiwan

We provide herein the correct structures of the compounds that were listed in Table 1 of the original paper.

## Figures and Tables

**Table 1:** Structures and IC_50_ values of 5-substituted 4,7-dimethoxy-1,3-benzodioxole derivatives for the inhibition of COLO 205 cells at 48 h.